# COVID-19 vaccine-associated liver injury and autoimmune hepatitis: a case series

**DOI:** 10.1097/MS9.0000000000004890

**Published:** 2026-04-02

**Authors:** Nida Mariyam, Mohammed Hady Albitar, Areez Shafqat, Rojina FathAlrahman, Hind Ibrahim Fallatah, Faisal Abaalkhail, Anas Ahmed Zahran, Abdullah AlMahmoud

**Affiliations:** a College of Medicine, Alfaisal University, Riyadh, Saudi Arabia; b Department of Medicine, King Abdulaziz University, Jeddah, Saudi Arabia; c Department of Liver & Small Bowel Transplantation & Hepatobiliary-Pancreatic Surgery, King Faisal Specialist Hospital & Research Center, Riyadh, Saudi Arabia; d College of Medicine, Ibn Sina National College for Medical Studies, Riyadh, Saudi Arabia; e Department of Internal Medicine, Gastroenterology, Dr. Soliman Fakeeh Hospital, Jeddah, Saudi Arabia

**Keywords:** autoimmune hepatitis, case series, COVID-19 vaccine, drug-induced liver injury, hepatology, liver injury

## Abstract

**Background:**

Coronavirus disease 2019 (COVID-19) vaccines have significantly reduced global morbidity and mortality. While generally safe, rare immune-mediated liver injuries, including autoimmune hepatitis (AIH) and drug-induced liver injury have been reported post-vaccination.

**Case series:**

We present three cases that developed liver injury following Pfizer-BioNTech (BNT162b2) COVID-19 vaccination. Case 1 describes a 64-year-old female with new-onset AIH confirmed by serology, histology, and clinical response to corticosteroids. Case 2 is a 36-year-old female who developed transient liver injury that resolved without immunosuppressive therapy. Case 3 involves a 35-year-old male with a history of AIH in remission who experienced a flare, requiring intensification of immunosuppressive treatment.

**Discussion:**

The mechanisms underlying liver injuries following COVID-19 vaccination remain incompletely understood, and a causal relationship has not been definitively established. One possible mechanism is molecular mimicry, in which the immune system targets liver cells after recognizing similarities with viral spike proteins. Vaccine adjuvants may also contribute to immune dysregulation in individuals with certain genetic factors like HLA-DR3 and HLA-DR4, which have been linked to AIH. Multiple reviews of vaccine-associated liver injury suggest that the majority of patients improve with immunosuppressive therapy, although there have been rare reports of severe presentations, including progression to liver failure.

**Conclusion:**

Although rare, COVID-19 vaccines may be associated with various forms of liver injury. This case series highlights the heterogeneous clinical spectrum and disease course of COVID-19 vaccine-associated liver injuries, ranging from transient liver enzyme abnormalities to more persistent disease ,such as autoimmune hepatitis, requiring treatment. While causality can not be established, these findings emphasize the importance of clinical vigilance and individualized assessment in at-risk populations.

## Introduction

COVID-19 vaccines have been adopted globally, supported by extensive clinical evidence demonstrating high efficacy^[^1^]^. Their administration has been accompanied by extensive safety surveillance programs, enabling continuous monitoring for rare or unexpected adverse events^[^1^]^. Immune-mediated liver injury has emerged as a rare but potential complication following coronavirus disease 2019 (COVID-19) vaccination^[^2^]^. Multiple studies have documented drug-induced liver injury (DILI) and new-onset or flares of autoimmune hepatitis (AIH) following administration of mRNA-based vaccines (Pfizer-BioNTech and Moderna mRNA-1273) and adenoviral vector-based vaccines (AstraZeneca AZD1222)^[^3,4^]^. Epidemiological studies have shown that COVID-19 vaccine-associated liver injury is exceedingly rare^[^5,6^]^. A pharmacovigilance study using Centers for Disease Control and Prevention/Vaccine Adverse Events Reporting System data found 53 reports of AIH following vaccination over a 2-year period, corresponding to approximately 0.21 cases per million vaccinated individuals (95% CI 0.16–0.27)^[^5^]^.

Affected patients commonly present with elevated liver enzyme levels, jaundice, and serological and histological features consistent with AIH. The majority of these patients respond favorably to corticosteroid therapy, similar to those with conventional AIH^[^7,8^]^. The aim of this case series is to present three cases of liver injury occurring after COVID-19 vaccination and to describe their clinical presentations, diagnostic features, and clinical course. This case series was reported in line with the PROCESS guideline^[^9^]^.

HIGHLIGHTS

- Autoimmune hepatitis (AIH) and transient liver injury have been rarely reported following COVID-19 vaccination, though the incidence remains extremely low (~0.21 per million).
- This case series describes three temporally associated liver presentations after COVID-19 vaccination: new-onset AIH, AIH flare, and possible transient liver dysfunction.
- Molecular mimicry and adjuvants are proposed mechanisms for vaccine-associated autoimmunity.
- Corticosteroids led to significant clinical and biochemical improvement in post-vaccination AIH cases.
- These cases underscore the need for post-vaccination monitoring and individualized risk assessment in high-risk patients and those with preexisting liver disease.

## Methods

### Case identification and selection

The three cases were identified as consecutive patients presenting to the hepatology clinic between January and August 2023 with new or worsening liver dysfunction occurring after administration of the COVID-19 vaccine. Patients were included if they had (1) new liver enzyme abnormalities or clinical jaundice within 60 days post-vaccination, (2) appropriate serological and radiological workup to exclude viral hepatitis and biliary obstruction, and (3) sufficient clinical data in the medical record to allow case characterization. Patients with another clear cause for liver injury (e.g., acute viral hepatitis, documented ischemic liver injury, or confirmed drug overdose) were excluded.

### Ethical approval and consent

Written informed consent for publication was obtained from all included patients. Institutional ethics approval was waived by the institutional review board because this retrospective case series involved review of routinely collected clinical data without any experimental intervention or alteration of patient management.

### Literature review approach

A literature search was performed through May 2024 using MEDLINE, PubMed Central, and Embase databases to identify relevant studies on liver injury and AIH associated with COVID-19 vaccination. Searches included combinations of terms such as COVID-19 vaccine, AIH, liver dysfunction, and DILI.

## Case series

### Case 1

A 64-year-old female presented with a 2-week history of jaundice, pruritus, nausea, vomiting, and right upper quadrant abdominal pain. Symptoms began within 24 hours of receiving the first dose of the Pfizer-BioNTech (BNT162b2) mRNA vaccine on 6 April 2023. The patient had a history of ischemic heart disease with a left ventricular ejection fraction of 35% and was taking aspirin, clopidogrel, bisoprolol fumarate, and perindopril. She had no known liver or autoimmune diseases and no relevant family history. On examination, the patient was afebrile and hemodynamically stable. Jaundice was present, but there was no abdominal tenderness or stigmata of chronic liver disease.

**Figure 1.**
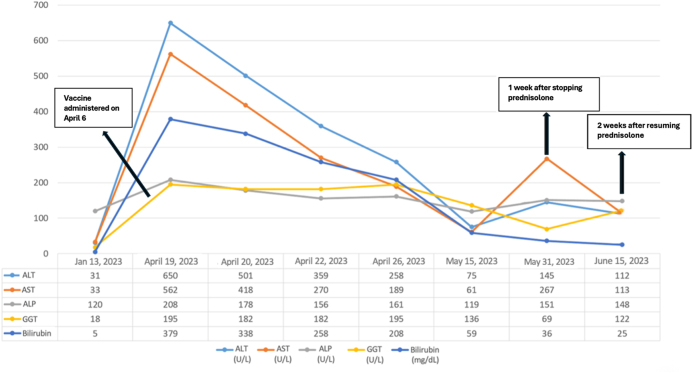
Liver enzyme levels for Case 1, showing improvement after treatment and the rise in levels after discontinuing medication. ALT, alanine aminotransferase; AST, aspartate aminotransferase; ALP, alkaline phosphatase; GGT, gamma-glutamyl transferase; bilirubin, total bilirubin. Dates on the *x*-axis represent calendar dates relative to vaccination.

Laboratory evaluation showed a mixed pattern of liver enzyme elevation, predominantly hepatocellular (Fig. 1). Other findings included: WBC: 13.5 × 10^9^/L, hemoglobin: 11.8 g/dL, platelet count: 336 × 10^9^/L, and INR: 1.1. Autoimmune workup showed a positive antinuclear antibody (ANA) titer of 1:320 with a speckled pattern, a weakly positive smooth muscle antibody (SMA), and an elevated IgG level of 17.5 g/L. The viral hepatitis panel was negative. Abdominal ultrasonography revealed a coarse hepatic echotexture and increased echogenicity, suggestive of acute hepatitis. Doppler study of the hepatic and portal veins was unremarkable. Liver biopsy revealed interface hepatitis with predominant lymphocytic infiltration and no evidence of fibrosis. According to the simplified IAIHG criteria, the patient scored 2 points for ANA positivity at a titer of 1:320 with weak SMA reactivity, 2 points for an IgG level exceeding 1.1 times the upper limit of normal, 2 points for exclusion of viral hepatitis, and 1 point for histology compatible with interface hepatitis, giving a total of 7 points, consistent with definite AIH^[^10^]^. She was started on prednisolone 20 mg daily, with minimal biochemical improvement after 2 weeks. At the 3-week follow-up, the dose was increased to 30 mg/day due to persistent hepatocellular enzyme elevation, resulting in significant clinical and biochemical improvement. After self-discontinuation of therapy, liver enzymes rose again, prompting reinitiation of prednisolone at 30 mg daily, which led to remission within 2 weeks. Following enzyme normalization, the dose was tapered by 5 mg every 1–2 weeks over 4–6 weeks, for a total treatment duration of approximately 10 weeks. She remained stable over the next 3 months of outpatient follow-up without relapse.

### Case 2

A 36-year-old previously healthy female presented with a 1-month history of progressive pruritus, jaundice, dark urine, and pale stools. She had received the first dose of Pfizer-BioNTech vaccine on 14 March 2023, 1 month before presentation. Pruritus began within hours of vaccination and gradually progressed over the following week to include jaundice, dark urine, and pale stools. She also reported mild epigastric discomfort, nausea, myalgia, and low-grade fever. The patient had no personal or family history of autoimmune or liver disorders. Physical examination revealed jaundice, widespread excoriations, and a palpable liver edge extending below the right costal margin.

**Figure 2.**
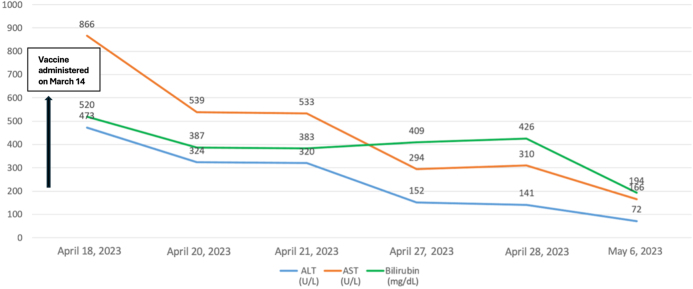
Liver function test results for Case 2, showing progressive decline in enzyme levels during supportive management. ALT, alanine aminotransferase; AST, aspartate aminotransferase; bilirubin, total bilirubin. Dates on the *x*-axis represent calendar dates relative to vaccination.

Initial laboratory evaluation demonstrated elevated alanine aminotransferase (ALT), aspartate aminotransferase (AST), ALP, GGT, and bilirubin (Fig. 2). WBC was 5.9 × 10^9^/L, hemoglobin 14.4 g/dL, and platelets 287 × 10^9^/L. Autoimmune serology revealed a positive ANA, an anti-SMA titer of 1:40, and a normal serum IgG level. Viral panels were negative for hepatitis A, B, and C, as well as for Cytomegalovirus, Epstein–Barr virus (EBV), and Varicella-zoster virus. Abdominal ultrasonography demonstrated a liver span of 14.3 cm with reduced echogenicity, periportal thickening, and starry sky appearance. No focal hepatic lesions were noted, and both portal and hepatic veins were patent. Doppler imaging excluded the presence of portal vein thrombosis. Liver biopsy was not done as the patient showed marked clinical improvement within the first 3 days of hospitalization with supportive care (Fig. 2).

She was administered intravenous hydration, ursodeoxycholic acid (UDCA) at 300 mg twice daily to reduce cholestasis, and cetirizine at 10 mg once daily to relieve pruritus. UDCA was started to manage clinically suspected cholestasis and pruritus, consistent with its established role in cholestatic liver disease. She was discharged after stabilization and followed up in the hepatology clinic after 1 week with full resolution of jaundice and pruritus. She was followed for approximately 4 weeks after discharge, during which liver enzyme levels remained normal without relapse. Longer-term follow-up was not available.

### Case 3

**Figure 3.**
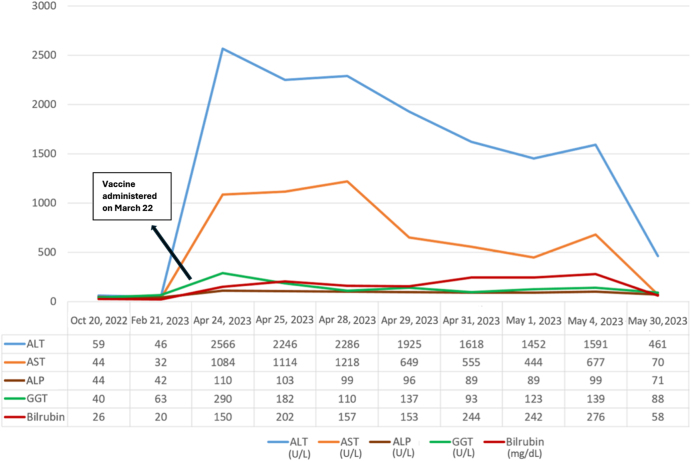
Liver function test results for Case 3, highlighting the fluctuation of liver enzymes during the flare-up. ALT, alanine aminotransferase; AST, aspartate aminotransferase; ALP, alkaline phosphatase; GGT, gamma-glutamyl transferase; bilirubin, total bilirubin. Dates on the *x*-axis represent calendar dates relative to vaccination.

A 35-year-old male with a history of AIH in remission presented with jaundice, nausea, fatigue, and epigastric pain 1 month after receiving the second dose of the Pfizer-BioNTech vaccine on 22 March 2023. He had been diagnosed with AIH for 15 years and had sustained biochemical remission with a dose of 2.5 mg prednisolone daily. Apart from known AIH, he had no additional personal or family history of autoimmune or liver disorders. The patient appeared dehydrated but was hemodynamically stable. There was no hepatosplenomegaly or abdominal distension, but marked jaundice was observed. Laboratory evaluation demonstrated a 10- to 20-fold elevation in AST and ALT, accompanied by mild–moderate increases in ALP, GGT, and total bilirubin (Fig. 3). The WBC count was 5.3 × 10^9^/L, hemoglobin was 14.7 g/dL, and platelet count was 158 × 10^9^/L. Autoimmune serology showed a moderately elevated ANA titer of 1:640, twice the baseline of 1:320 documented 5 years earlier. The serum IgG level was 12.1 g/L compared with the baseline level of 22.6 g/L. Viral screening was negative for hepatitis A, B, and C, as well as for EBV.

Radiological evaluation was not performed as the clinical presentation and laboratory findings were sufficient to diagnose an AIH flare, and there were no signs of abdominal distension, focal tenderness, or biliary obstruction. Given concern for a biochemical flare of underlying AIH, the prednisolone dose was increased to 1 mg/kg daily. Liver enzymes gradually improved over 4 weeks, after which the dose was tapered by 10 mg weekly and returned to the prior maintenance dose of 2.5 mg daily. The total duration of intensified corticosteroid therapy was approximately 8 weeks. Liver biopsy was not repeated because the patient had long-standing biopsy-proven AIH and was presented with a characteristic biochemical flare that responded to intense immunosuppression, so additional biopsy was not clinically indicated. He remained stable on 2.5 mg/day prednisolone with no further flares reported during 4 months of follow-up.

## Discussion

The reported cases highlight the variable clinical and biochemical patterns of liver injury associated with COVID-19 vaccination. Case 1 met the simplified diagnostic criteria for AIH, with histological confirmation and steroid responsiveness^[^10^]^. These features make new-onset AIH temporally associated with vaccination a reasonable consideration. However, the possibility that this represents spontaneous AIH coinciding with vaccination should be considered, as the reported 24-hour latency is unusually short for new-onset AIH, which typically develops through delayed adaptive immune mechanisms. Most reported cases of vaccine-associated AIH present with a latency of approximately 10–14 days after vaccination. However, in rare instances, onset has been documented as early as 2–3 days (including rare reports up to 1 day) after vaccination, which calls into question the mechanism of vaccine-associated new-onset AIH^[^11,12^]^.

In Case 2, SMA was weakly positive at a titer of 1:40, a level insufficient to support a diagnosis of AIH, and the biochemical abnormalities resolved spontaneously with supportive management, which might be consistent with transient DILI^[^8^]^. However, the latency of nearly 1 month after vaccination might be more compatible with possible vaccine-associated liver injury rather than vaccine-induced DILI. Although viral studies were negative and no new medications were reported, transient hepatitis related to an unrecognized viral illness or other drug exposure cannot be fully excluded, particularly given the spontaneous improvement with supportive care alone. Overall, this case is best interpreted as a transient liver injury temporally associated with vaccination rather than confirmed vaccine-induced DILI. In Case 3, the AIH flare after more than 10 years of remission also responded to immunosuppression, suggesting a potential vaccine-associated trigger. Although the flare occurred approximately 1 month after vaccination, this temporal association alone is insufficient to confirm that vaccination triggered the relapse. Given the patient’s known history of AIH, low-dose maintenance prednisolone, serologic findings (ANA 1:640 with IgG lower than prior baseline), and the clinical course, there is a possibility that the episode may be explained by a spontaneous relapse rather than a de novo, vaccine-induced injury. Table 1 summarizes the demographic characteristics, clinical presentation, and outcomes of the three reported cases.

**Table 1:** Summary of demographics, vaccination details, patient background, clinical presentation, investigations, treatment, and outcomes for all three cases.

Variable	Case 1	Case 2	Case 3
Age/sex	64/F	36/F	35/M
Comorbidities/regular medications	Ischemic heart disease; aspirin, clopidogrel, bisoprolol, perindopril	None	AIH in remission; prednisolone 2.5 mg
Vaccine type and dose	Pfizer-BioNTech (BNT162b2), Dose 1	Pfizer-BioNTech (BNT162b2), Dose 1	Pfizer-BioNTech (BNT162b2), Dose 2
Latency to symptom onset (days)	~1 day	<1 day pruritus; ~7 days jaundice	~30 days
Presenting symptoms	Jaundice, RUQ pain, pruritus, nausea/vomiting	Jaundice, pruritus, pale stool, dark urine, fever	Jaundice, fatigue, nausea
IgG	17.5 g/L	Normal	12.1 g/L (baseline 22.6 g/L)
Autoimmune serology	ANA 1:320; SMA weakly positive	ANA positive; SMA 1:40	ANA 1:640
Biopsy and findings	Yes – interface hepatitis, lymphocytic infiltrate	No	No
Diagnosis	New-onset AIH	Self-limited DILI/transient liver injury	AIH flare
Treatment	Prednisolone 20 mg, increased to 30 mg, then tapered	Supportive care; UDCA; antihistamine	Prednisolone increased to 1 mg/kg
Taper	5 mg every 1–2 weeks	–	10 mg every 1–2 weeks, then 5 mg every 1–2 weeks
Clinical course	Relapse after self-discontinuation of steroids; followed by full remission	Full resolution	Gradual improvement over 4 weeks

ANA, antinuclear antibody; SMA, smooth muscle antibody; IgG, immunoglobulin G; RUQ, right upper quadrant; DILI, drug-induced liver injury; AIH, autoimmune hepatitis.

Although only a few cases of COVID-19 vaccine–associated DILI and AIH have been reported, our findings add to the limited existing literature, and the clinical variability observed in our cases aligns with previously described patterns of liver injury following COVID-19 vaccination^[^11–13^]^. In a recent systematic review, a range of COVID-19 vaccine-associated hepatic adverse events were identified, with AIH being the most frequently reported, followed by portal vein thrombosis, elevated transaminase levels, and other liver-related complications, including DILI^[^13^]^. In the reported cases, women over the age of 50 years were most frequently affected, with symptoms typically presenting 10–14 days post-vaccination^[^11,14^]^. Diagnosis is typically made within 3 weeks of vaccination, with jaundice, elevated IgG, and positive ANA and SMA as the most common findings^[^14–16^]^. In our case series, autoantibody findings were likewise heterogeneous, ranging from high-titer ANA with elevated IgG to weaker serologic positivity, consistent with patterns described in prior reports. ANA is a nonspecific marker and may be present in healthy individuals; thus, diagnostic interpretation should always incorporate IgG levels, histological assessment when available, exclusion of viral hepatitis, and the patient’s clinical course.

COVID-19 vaccines are developed using multiple platforms capable of inducing varying immune responses, including systemic and mucosal immunity, as described in studies of intranasal formulations^[^17^]^. The optimal immunosuppressive therapy for vaccine-associated AIH remains unclear; however, corticosteroids remain the mainstay of treatment^[^8,10^]^. Most patients achieve biochemical and clinical resolution and have an excellent prognosis^[^14^]^. In a multicenter cohort of 47 patients, 41 required immunosuppression, 1 required transplantation, and another died of acute liver failure; however, the remaining patients recovered without long-term morbidity^[^14^]^. In a review of 5041 cases of vaccine-associated liver injury, most achieved clinical and biochemical resolution with immunosuppression. Three of these cases showed severe disease progression, including liver failure, transplantation, and death^[^18^].^ Similarly, a systematic review by Chow *et al* examined 32 cases of AIH-like syndromes following COVID-19 vaccination. Jaundice was reported in 81% of these patients, and 75% were treated with corticosteroids. Overall, 97% of patients showed improvement or full resolution of symptoms^[^12^]^. Consistent with larger case series and systematic reviews, our cases showed a variable clinical course, ranging from self-limited disease to presentations requiring corticosteroid therapy. Management was individualized, with corticosteroids required in Cases 1 and 3 in the setting of persistent hepatocellular enzyme elevation accompanied by autoimmune features, whereas Case 2 followed a milder course and was managed conservatively. The observed latency ranged from within 24 hours to approximately 1 month after vaccination, and all patients achieved favorable clinical and biochemical outcomes during follow-up. Biochemical remission was achieved with corticosteroid therapy in Cases 1 and 3 within approximately 8–10 weeks, while Case 2 experienced spontaneous normalization of liver enzymes within 4 weeks with supportive management. No patient developed clinical deterioration, suggesting liver failure, during the reported follow-up period.

The development of immune-mediated liver injury following COVID-19 vaccination remains poorly understood. Host-specific factors, including age, comorbidities, prior antigen exposure and genetic predisposition, have been shown to influence vaccine responses and may partially explain why only rare individuals experience immune-mediated sequelae following immunization^[^19^]^. Molecular mimicry, in which viral spike proteins or vaccine adjuvants trigger an aberrant immune response in genetically susceptible individuals, has been proposed as a potential mechanism in some studies^[^19^]^. Additionally, other studies have suggested that adjuvants in vaccines that enhance the immune response may contribute to the dysregulation of the immune system in genetically predisposed individuals^[^20^]^. Vaccine-associated liver injury may also be influenced by genetic predispositions such as specific human leukocyte antigen alleles associated with AIH^[^21^]^. In a review by Wang *et al*, which analyzed 35 reported cases of AIH following COVID-19 vaccination, including vaccines such as AstraZeneca, Moderna, and Pfizer-BioNTech, several patients were found to carry specific human leukocyte antigen alleles (HLA-DR3 or HLA-DR4), both of which are strongly linked to classical AIH^[^17,21^]^. Although HLA typing was not conducted in our patients, some studies suggest that host-specific immunogenetic factors may influence susceptibility; therefore, any such contribution in this series remains speculative. Future studies incorporating HLA typing may help clarify whether specific immunogenetic factors contribute to susceptibility.

Recent data suggest that mRNA-based COVID-19 vaccines, including Pfizer-BioNTech and Moderna, may be more commonly linked to autoimmune complications than traditional vaccines^[^7,22^]^. This observation highlights the need to explore alternative vaccine formulations for individuals with preexisting autoimmune conditions. Protein-based or inactivated viral vaccines may pose a lower risk of immune system dysregulation. It is unclear whether boosters increase the risk of liver injury. In an observational study of 47 patients with elevated liver transaminases within 90 days of vaccination, 17 patients received the first dose, 22 patients received the first booster dose, and 8 patients received the second booster dose^[^14^].^ Liver injury appeared to be more severe after the booster administration than after the primary dose. Notably, 28% of the patients had mild relapse after boosters. Immunosuppression at re-administration significantly lowered relapse rates (28.6 vs. 88.9%; *P* = 0.007)^[^14^]^. These findings suggest a role for preemptive corticosteroid therapy in selected patients. However, current evidence is insufficient to support routine preemptive corticosteroid use, and further prospective studies are required.

Studies largely agree that vaccine-associated liver injury does not pose a significant safety concern relative to the benefit of vaccines in preventing severe COVID-19 in at-risk populations^[^23^]^. Our cases underscore the variable presentations and the need for clinical vigilance regarding this rare but important adverse event. Larger, multicenter studies with more diverse populations are essential to fully elucidate the genetic and environmental factors influencing susceptibility to vaccine-associated liver injury. In addition, it is important to evaluate the long-term effects of such injuries, particularly in individuals who require long-term immunosuppressive therapy.

This study has some limitations. First, the small sample size limits the generalizability of the findings. Second, since biopsy with histopathological confirmation was obtained only in Case 1, the diagnosis of the remaining cases was based primarily on clinical and serologic features. Third, HLA typing was not performed, which could have provided valuable insights into the potential genetic susceptibility factors contributing to the observed autoimmune response, as suggested in some reports. Fourth, since no structured causality tool (e.g., RUCAM) was used, the association between vaccination and liver injury remains insufficient to confirm causality. Despite these limitations, the cases presented offer meaningful contributions to the understanding of the clinical variability and outcomes associated with COVID-19 vaccine-associated liver injury.

## Conclusion

This case series contributes to the evolving understanding of the diverse clinical presentations of liver injury associated with COVID-19 vaccination. While these cases highlight potential temporal associations, they do not fully establish a causal relationship between vaccination and hepatic injury. Although the overall incidence of these complications is exceedingly rare given the scale of global vaccination, clinicians should remain vigilant for the possibility of hepatic complications, especially in individuals with preexisting liver disease or underlying immune dysfunction. Post-vaccination monitoring and individualized risk assessments for at-risk individuals, such as those with liver dysfunction or other autoimmune conditions, remain essential to ensure early diagnosis and timely management of potential vaccine-associated AIH and other hepatic complications.

## Data Availability

All data generated or analyzed during this study are included in this published article.
